# In Vitro Evaluation of Rosemary Essential Oil: GC-MS Profiling, Antibacterial Synergy, and Biofilm Inhibition

**DOI:** 10.3390/ph17121653

**Published:** 2024-12-08

**Authors:** Daniel E. K. Kabotso, David Neglo, Sarah E. Gaba, Emmanuel K. Danyo, Alberta D. Dayie, Anastasia A. Asantewaa, Fleischer C. N. Kotey, Nicholas T. K. D. Dayie

**Affiliations:** 1 Department of Basic Sciences, School of Basic and Biomedical Sciences, University of Health and Allied Sciences, PMB 31, Ho 00233, Ghana; ekabotso@uhas.edu.gh (D.E.K.K.); dneglo@uhas.edu.gh (D.N.); 2 Department of Biomedical Sciences, School of Basic and Biomedical Sciences, University of Health and Allied Sciences, PMB 31, Ho 00233, Ghana; esinamgaba@gmail.com; 3 Department of Technologies for Organic Synthesis, Institute of Chemical Technology, Ural Federal University, Mira Street 28, 620002 Ekaterinburg, Russia; e.kdanyo@gmail.com; 4 Department of Chemistry, University of Cape Coast, Cape Coast 00233, Ghana; alberta.dayie@ucc.edu.gh; 5 Department of Medical Microbiology, University of Ghana Medical School, Korle Bu, Accra 00233, P.O. Box KB 4236, Ghanafcnkotey@flerholiferesearch.com (F.C.N.K.)

**Keywords:** *Rosmarinus officinalis*, essential oil, antibacterial activity, *Escherichia coli*, *Klebsiella pneumoniae*, MRSA, *Streptococcus mutans*, *Bacillus subtilis*, *Pseudomonas aeruginosa*, *Salmonella* Typhi

## Abstract

**Background:** Antimicrobial resistance (AMR) has become precarious, warranting investments in antimicrobial discovery. **Aim:** To investigate the antibacterial activity of rosemary essential oil (REO), alone and in combination with selected conventional antibiotics. **Methods:** REO was subjected to antimicrobial susceptibility testing (including minimum bactericidal concentration (MBC) and minimum inhibitory concentration (MIC) determination) and investigation of anti-pre-biofilm and antibiofilm activities. **Results:** The phytochemical composition of the REO was eucalyptol (42.68%), bornanone (33.20%), endo-borneol (9.37%), α-terpeneol (7.95%), linalool (2.10%), bornyl acetate (1.81%), caryophyllene (1.09%), 4-terpeneol (0.94%), and anethole (0.87%). The antibacterial inhibition zones generally increased with increasing REO concentration (i.e., 10, 20, 50, 100, and 200 mg/mL). The MIC and MBC ranges of REO for all bacteria were 3.13–6.25 mg/mL and 3.12–12.5 mg/mL, respectively. The MICs (in µg/mL) of ciprofloxacin, chloramphenicol, streptomycin, tetracycline, and ampicillin, respectively, were *Escherichia coli* (0.98, 3.92, 1.96, 7.81, and 250), *Klebsiella pneumoniae* (1.25, 7.81, 125, 7.81, and 1000), MRSA (62.5, 7.81, 3.91, 7.81, and 250), *Streptococcus mutans* and *Bacillus subtilis* (125, 15.68, 250, 31.25, and 1000), *Pseudomonas aeruginosa* (125, 31.25, 500, 31.25, and 1000), and *Salmonella* Typhi (0.98, 15.68, 125, 1.96, and 1000). The MBC-MIC ratios of REO against all bacteria were in the range 1–2, indicating bactericidal effects. Mainly synergy (FICI = 0.16–0.37) was observed between REO and the conventional antibiotics. The IC50 values (in µg/mL) of REO against the bacteria, pre-biofilm vs. biofilm formation, were *E. coli* (1342.00 vs. 4.00), *K. pneumoniae* (106.00 vs. 3.00), MRSA (134.00 vs. 6.00), *S. mutans* (7259.00 vs. 7.00), *B. subtilis* (120.00 vs. 7.00), *P. aeruginosa* (4989.00 vs. 7.00), and *S.* Typhi (10.00 vs. 2.00). **Conclusions:** Rosemary essential oil had significant bactericidal effects on the bacteria tested, and its MIC and MBC values were low. Overall, it was synergistic with known conventional antibiotics and, thus, has encouraging prospects in combination therapy involving conventional antibiotics, even in the treatment of infections with multidrug-resistant bacteria, including biofilm-forming ones.

## 1. Introduction

Antimicrobial resistance (AMR), which had its onset shortly after the discovery of the first antibiotic, penicillin, has become more precarious in the 21st century, creating a seemingly perpetual global public health crisis [1]. To illustrate, it accounted for about 5 million deaths in 2019 alone and can result in an annual death toll of double this figure by 2050 [1,2,3]. While antimicrobial stewardship efforts have consequentially been intensified to curtail the anthropogenic drivers of AMR, they are not enough to obliterate the AMR menace. It is imperative to concurrently complement these efforts with investments in sustainable discovery of newer antimicrobials, such as from plant sources [4,5].

One plant that has a huge potential for exploitation in antimicrobial chemotherapy is *Rosmarinus officinalis* L., which is commonly referred to as rosemary [6,7,8]. Historically, its leaves and essential oils have been used in the preservation and spicing of food as well as in alternative medicine [9]. Furthermore, phytochemical analyses of its essential oils have demonstrated the presence of such bioactive agents as 3-carene, pinenes, camphene, camphor, and 1,8-cineole, which account for its antimicrobial activity [10,11,12]. There have also been sporadic reports on its antimicrobial effects, such as against bacteria like *Listeria monocytogenes*, *Klebsiella pneumoniae*, *Pseudomonas aeruginosa*, *Staphylococcus aureus*, and *Salmonella* Typhimurium [7,8,10,13].

One key blind spot of investigations on the antibacterial activity of rosemary essential oil (REO) is the general absence of data on the exploration of its synergistic effects when combined with conventional antibiotics. In addition, these studies hardly originate from sub-Saharan Africa, where the bulk of the AMR problem is felt [1]. Meanwhile, AMR patterns of bacterial strains are geographically dynamic, as is the distribution of the various bioactive compounds within the essential oils. To help clarify the “blind spot” and provide further insights, this study investigated the antimicrobial activity of REO, alone and in combination with selected conventional antibiotics, using REO extracted from rosemary plants in Ghana, one of the AMR hotspots in sub-Saharan Africa.

## 2. Results

### 2.1. Gas Chromatographic–Mass Spectrometric-Based Phytochemical Analysis of REO

The phytochemicals present in REO, based on gas chromatography, were diverse, and their relative proportions decreased across eucalyptol (42.68%), bornanone (33.20), endo-borneol (9.37%), α-terpeneol (7.95%), linalool (2.10%), bornyl acetate (1.81%), caryophyllene (1.09%), 4-terpeneol (0.94%), and anethole (0.87%). Further details of the gas chromatographic analysis are presented in Table 1.

### 2.2. Susceptibility of Selected Bacterial Pathogens to Varying Concentrations of REO

Generally, there was a trend of increasing size of the inhibition zone with an increasing concentration of REO for the bacteria tested, even for MRSA. In the case of *K. pneumoniae*, of the five concentrations of REO examined (10 mg/mL, 20 mg/mL, 50 mg/mL, 100 mg/mL, and 200 mg/mL), only the highest tested REO concentration—200 mg/mL—demonstrated an inhibitory effect. Regarding *S. mutans*, the inhibitory effects were observed starting with the 50 mg/mL REO concentration, whereas in the other bacteria tested—*E. coli*, MRSA, *B. subtilis*, *P. aeruginosa*, and *S.* Typhi—inhibitory effects were observed even at the lowest REO concentration tested (10 mg/mL). Details of the susceptibility of various bacterial pathogens to varying concentrations of REO are presented in Table 2 and Figure S1.

### 2.3. Minimum Inhibitory and Bactericidal Concentrations of REO Against the Bacterial Pathogens

Table 3 details out the minimum inhibitory concentration (MIC) and the minimum bactericidal concentration (MBC) of REO and a range of conventional antibiotics against various strains of bacteria. *B. subtilis*, *P. aeruginosa*, and *S.* Typhi had the lowest MIC values for REO (3.13 mg/mL), while *B. subtilis* and *P. aeruginosa* had the least MBC values (3.12 mg/mL). Overall, the MIC values ranged from 3.13 mg/mL to 6.25 mg/mL, and the MBC values ranged from 3.12 mg/mL to 12.5 mg/mL. The ratio (R) between the MBC and the MIC, which informs on whether the effect is bactericidal (R ≤ 4) or bacteriostatic (R > 4) recorded against each of the bacterial pathogens, shows that REO was bactericidal against all the bacteria tested.

### 2.4. Synergistic Effects of Various REO–Conventional Antibiotic Combinations Against a Range of Bacterial Pathogens

The synergistic effect of REO, when combined with various antibiotics, is presented in Table 4. The fractional inhibitory concentration index (FICI), which is used to interpret the effect (values 0.5 representing synergism, >0.5 and 1 representing partial synergism, =1 representing an additive effect, >1 and 4 representing indifference or no difference, and >4 representing antagonism), showed that for most of the bacteria, the combination of REO with various antibiotics had synergistic or partially synergistic effects.

### 2.5. REO Inhibition of Pre-Biofilms and Biofilms of Various Bacterial Pathogens

Table 5 presents the IC50 values for REO’s inhibition of pre-biofilms and biofilms of various bacteria. There were lower IC50 values for biofilm formation, indicating that REO is more effective in inhibiting biofilm formation than it is in inhibiting pre-biofilm formation for all the tested bacterial pathogens.

**Figure 1:**
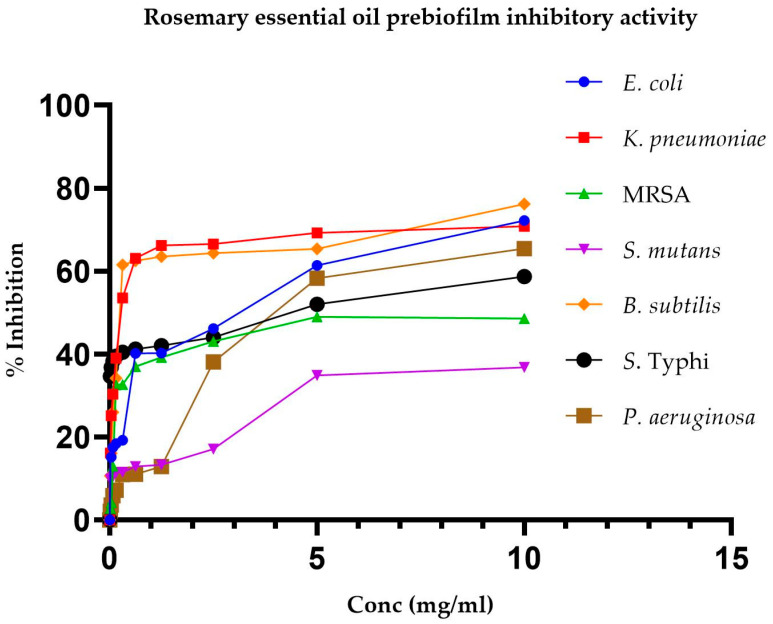
Inhibition of pre-biofilm formation potential of different bacterial strains by rosemary essential oil.

**Figure 2:**
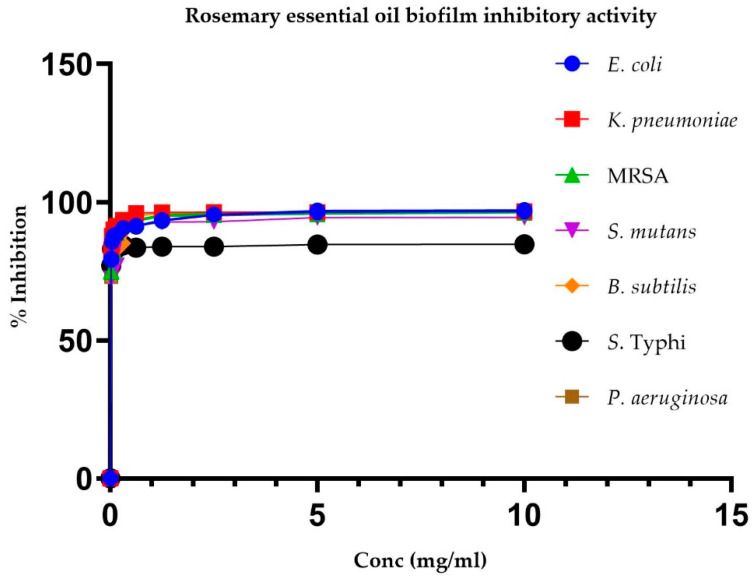
Inhibition of biofilm formation potential of different bacterial strains by rosemary essential oil.

Moreover, Figure 1 and Figure 2 depict the changes in percentage inhibition with increasing REO concentration for pre-biofilms and biofilms, respectively.

## 3. Discussion

The increased precariousness of AMR calls for an intensified exploration for newer antimicrobials, especially from plant sources [1,4,5]. To that end, this study investigated the antimicrobial activity of REO extracted from rosemary plants in Ghana, alone and in combination with selected conventional antibiotics, against a spectrum of bacteria, multidrug-resistant ones inclusive.

As previously highlighted, the REO used in the investigation in the current study was mainly composed of eucalyptol and bornanone, with the other constituents of the REO—endo-borneol, α-terpeneol, linalool, bornyl acetate, caryophyllene, 4-terpeneol, and anethole—being in the minority. In contrast, in the studies of Jiang et al. [10], Dhouibi et al. [14], and Murtiastutik et al. [15], 1,8-cineole, camphor, and α-pinene were the dominant constituents. In the study of Becer et al. [16] as well, these three compounds were in the majority, alongside verbenone. Interestingly, none of these studies detected the compounds that dominated in the REO used in the current study, i.e., eucalyptol and bornanone. It is noted, though, that bornyl acetate and caryophyllene, which were detected at lower levels in the current study, were also detected by Jiang et al. [10], Murtiastutik et al. [15], and Becer et al. [16]. These disparities in phytochemical distribution may be accounted for by variations in sources of the REO as well as the extraction method, as alluded to by Melero-Bravo et al. [17].

The diversities in REO phytochemical constituents notwithstanding, significant antibacterial activity of REO was observed against the bacteria tested in this study—*E. coli*, *K. pneumoniae*, MRSA, *S. mutans*, *B. subtilis*, *P. aeruginosa*, and *S*. Typhi—consistent with previous reports on REO. Examples are Alvarez et al.’s [7] and Garcia-Sotelo et al.’s [8] reported significant REO inhibition of *Salmonella* as well as those reported by Puvača et al. [18] against *S. aureus*, Araya et al. [19] against *E. coli* and *S. aureus*, Eid et al. [13] against MRSA, *K. pneumoniae*, and *P. aeruginosa*, Becer et al. [16] against *E. coli*, *K. pneumoniae*, and *S. aureus*, and that of Dhouibi et al. [14] against *E. coli*, *P. aeruginosa*, *Salmonella enterica*, and *S. aureus*. Expectedly, there was a trend of increasing size of the inhibition zone with increasing concentration of REO for these bacteria. Interestingly, even at the lowest concentration of REO tested, MRSA and *P. aeruginosa* were inhibited, which only underscores the promise that REO holds in future antimicrobial chemotherapy against multidrug-resistant pathogens.

The prospects of REO in infectious disease treatment are further deepened by the observed low MIC and MBC values for REO. Similarly, Alvarez et al. [7], Garcia-Sotelo et al. [8], and Puvača et al. [18] also reported low MIC levels of REO against *Salmonella cholerae* and *Listeria monocytogenes*, *Salmonella typhimurium* and *Listeria monocytogenes*, and *S. aureus*, respectively. Moreover, the ratio (R) between the MBC and the MIC, which informs on whether the effect recorded against the bacterial pathogens is bactericidal (R ≤ 4) or bacteriostatic (R > 4), demonstrated the REO to be bactericidal, further increasing its therapeutic value.

Although it is difficult to tell the mechanism of action of REO from the data generated in the current study, it has been noted elsewhere that REO is able to exploit protein transport and ion channels via which it enters the cytoplasm [20]. An alternative mechanism involves disrupting the cell membrane to impede ion transport and cell membrane respiration [20]. Although it can be argued that these mechanisms may be more applicable to fungal pathogens, it goes to reason that the bactericidal effects of REO may be orchestrated through a similar strategy. It may also have demonstrated its antibacterial activity (by virtue of its phytochemicals) through (a) disruption of cell wall synthesis, (b) induction of oxidative stress (such as via protein oxidation, DNA/RNA damage, antioxidant depletion, or reactive oxygen species generation), (c) protein denaturation (for example, via cellular stress, metabolic disruption, structural protein damage, or enzyme inactivation), (d) membrane damage (such as via component leakage, structural integrity loss, increased permeability, or phospholipid bilayer disruption), (e) efflux pump interference, (f) biofilm disruption, (g) quorum sensing interference, or (h) combinations thereof, in the tested bacteria [21,22,23,24]. Further analyses on REO can clarify insights on its mechanism(s) of action, as well as its potential acute and sub-chronic toxicity to humans and animals at therapeutic levels.

One welcomed observation in the current study was the demonstrated synergistic effects of REO-conventional antibiotic combinations against most of the bacterial pathogens tested. Although this seems to be the first report suggesting successful synergy between REO and conventional antibiotics, it is noted that synergistic effects between REO and other essential oils have been previously demonstrated [20,25,26,27,28]. Of course, such essential oil–essential oil synergy can be complicated by variations in chemical compositions of the given essential oils involved [27]. That said, the synergistic effects observed in this study highlight the potential flexibility of REO use in combination with conventional antibiotics (whose solitary use may have otherwise resulted in treatment failure) in the treatment of infectious diseases. It also reduces the likelihood of imminent AMR development against REO.

Also worth noting is the observed effectiveness of REO in inhibiting biofilms of the test bacterial strains by appreciable dose-dependent concentration of the test extract showing very good IC50s—the lower the IC50s values, the better the biofilm–inhibitory activity. Microbial biofilms are known to cause resistant chronic infections [29]. This activity of the extract in many studies have been attributed to the potential of the active components in the test extract impairing the adhesion of planktonic cells on abiotic surfaces, reducing biofilm metabolic activity, or promoting biomass degradation of mature biofilms as a major breakthrough [30].

This study was limited, albeit negligibly, by the inability of the oil and the broth to evenly mix in the wells, and, hence, the activities recorded may have been underestimated.

## 4. Materials and Methods

### 4.1. Materials

Mueller-Hinton broth (Oxoid, CM045), Mueller-Hinton agar (Oxoid CM0337), and nutrient agar (Oxoid, CM0003) were purchased from Oxoid Ltd., Wade Road, Basingstoke, Hants, RG24 8PW, UK. Tetrazolium salt, TTC, was bought from CDH (Central Drug House (P) Ltd., Cop. Office, 7/28 Vardam House, Daryagang, Delhi-110002, India). Anhydrous sodium sulfate (Daejung Chemicals and Metals CO., Ltd., 186 Seohaean-ro, Siheung-si, Gyeonggi-do, Korea), dimethyl sulfoxide, and MTT dye were purchased from the local vendor Wallart Lab Enterprise (No. 12 Konkonte (Akotex) Street, Kokomlemle, behind Ring Road Central Access Bank, Accra, Ghana). An AKAI high-performance commercial blender was purchased from MELCOM at Stadium Road, Ho, Volta Region, Ghana. Sterile 96-well microtitre plates were purchased from China (Citotest Labware Manufacturing Co. Ltd., Jiangsu, China). Standard antibiotics such as ciprofloxacin, chloramphenicol, streptomycin, tetracycline, and ampicillin were purchased from the local vendor Benburto Enterprise Ltd. (144 Dansoman High St., Sakaman-Accra, Ghana).

### 4.2. Test Microorganism

Bacterial isolates such as *Escherichia coli* (ATCC 25922), *Pseudomonas aeruginosa* (ATCC 4853), *Klebsiella pneumoniae* (NCTC 13440), methicillin-resistant *S. aureus* (MRSA) (NCTC 12493), *Streptococcus mutans* (ATCC 700610), *Bacillus subtilis* (ATCC 10004), and *Salmonella* Typhi (ATCC 14028) were obtained from the Microbiology Unit of the School of Basic and Biomedical Science Laboratory of the University of Health and Allied Sciences, Ho, Ghana. These microorganisms were sub-cultured for 24 h prior to the experiment, on nutrient agar, at 37 °C.

### 4.3. Study Site

This study was conducted in the Analytical and Organic Chemistry Laboratory of the Department of Basic Sciences and Microbiology Laboratory of the Department of Biomedical Sciences in the School of Basic and Biomedical Sciences at the University of Health and Allied Sciences, Ho, Volta Region, Ghana.

### 4.4. Study Design

The study employed an experimental research design. The tests were conducted in duplicates to obtain reliable results, and control groups were also used to validate the results from experimental groups.

### 4.5. Collection of Samples

Plant samples were bought from the Ho Central Market in the Volta Region of Ghana. Diseased and unfit leaves were not sampled. These samples were authenticated by an experienced botanist and assigned the herbarium voucher number “UHAS/ITAM/2024/L015”.

### 4.6. Extraction of Rosemary Essential Oil

**Figure 3:**
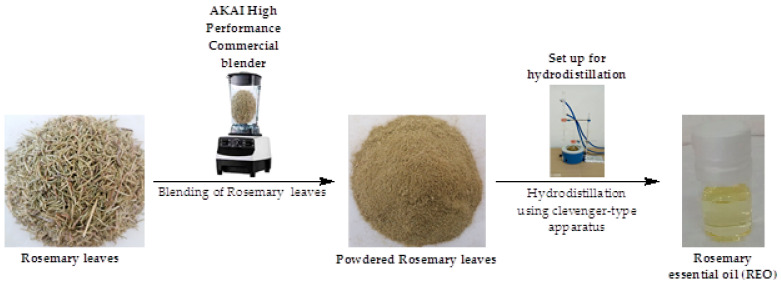
Setup for the extraction of REO using a Clevenger-type apparatus.

The rosemary leaves were blended into powder using the AKAI high-performance commercial blender; 80 g of the powdered rosemary leaves was suspended in 300 mL of distilled water in a 300 mL round bottom flask and subjected to hydrodistillation for three hours using a Clevenger-type apparatus (Figure 3). The condensed vapors of the oil and water were drained through the delivery tube of the Clevenger-type apparatus and the oil layer separated from the water layer using a 25 mL separating funnel. The separated REO was dried over anhydrous sodium sulfate, transferred into a 10 mL vial, and capped. The vial was wrapped in aluminum foil and then stored at 4 °C until analysis [31].

The percentage yield of REO (2.96 g) from rosemary powder (80.0 g) was calculated to be 3.70% by using the following formula:

Percentage Yield=Massofextractedrosemaryessentialoil, REO (g)Massofpowderedrosemaryleaves (g)×100%

### 4.7. Determination of Stock Concentration and Preparation of Various Concentrations of REO

One milliliter (1.0 mL) of REO was weighed on an analytical balance thrice to determine its stock concentration as 953 mg/mL. The stock REO was used to prepare various diluted concentrations (10–200 mg/mL) by using the dilution formula *C*_1_*V*_1_ = *C*_2_*V*_2_, where *C*_1_ is the initial concentration in mg/mL, *C*_2_ is the final concentration in mg/mL, *V*_1_ is the initial volume in ml, and *V*_2_ is the final volume in ml. Dimethyl sulfoxide was employed as the solvent [31].

### 4.8. Phytochemical Analysis via GC-MS

Aliquots of REO (50 µL) were taken up in hexane (50 µL), mixed and readied. The analysis of REO was performed using a GC Clarus 580 gas chromatograph interfaced with a Clarus SQ 8 mass spectrometer (PerkinElmer Inc., Waltham, MA, USA). The resolution was achieved using a ZB-5HTMS (5% diphenyl/95% dimethyl polysiloxane, Phenomenex Inc, Torrance, CA USA) fused capillary column (30 m length × 0.25 μm ID × 0.25 μm film thickness). The programming was as follows: Initial temperature was 40 °C (isothermal for 2 min), then increased at 10 °C/min to 250 °C, then 20 °C/min to 280 °C, and held for 10 min at 280 °C. Helium gas (99.9999%: Air Liquide, Tema, Ghana) was used as a carrier gas at a constant flow rate of 1 mL/min, and an injection volume of 1 μL was used. The solvent delay was 0 to 3 min, and the total GC/MS running time was 34.5 min. For GC/MS detection, an electron ionisation system was operated in electron impact mode with an ionisation energy of 70 eV. The injector temperature was maintained at 250 °C, and the ion-source temperature was 220 °C. Mass spectra were taken at 70 eV at a scan interval of 1s and fragments from 50 to 450 Da. The mass detector was Turbo-Mass TM, and the software adopted to handle mass spectra and chromatograms was Turbo-Mass ver. 6.1.0. (PerkinElmer Inc.). Compounds were identified according to the NIST 2014 mass spectral library, which is a standard MS reference database released by the National Institute of Standards and Technology (NIST).

### 4.9. Determination of Antibacterial Susceptibility

Mueller-Hinton agar (20 mL) was aseptically dispensed into Petri dishes and allowed to solidify. The EUCAST (European Committee on Antimicrobial Susceptibility Testing) Standard was followed. Briefly, overnight cultures at a final concentration of 10^8^ CFU/mL using a 0.5 McFarland standard were inoculated onto the Mueller-Hinton agar using sterile swab sticks. Each organism was inoculated on a different plate and then labelled accordingly. They were subsequently left to dry for fifteen minutes. Seven wells were bored in each plate using a cork borer having a diameter of 6 mm. Next, 100 µL of rosemary essential oil at concentrations of 200, 100, 50, 20, and 10 (mg/mL) was dispensed into each well on separate plates. One well was filled with 20% DMSO in 0.5% Tween 80 as a negative control. Chloramphenicol (30 µg) was placed in the seventh well as the positive control, owing to its broad-spectrum activity. The extracts were allowed to diffuse for 15 min at room temperature after which they were incubated at 37 °C for 24 h for bacterial activity. After incubation, the plates were checked for the formation of clear zones around the well, which corresponds to the antimicrobial activity of the essential oil; the zones of inhibition (ZOIs) were measured using a rule and recorded in mm [32]. The procedure was performed in duplicates [33,34].

### 4.10. Determination of Minimum Inhibitory and Bactericidal Concentrations

The minimum inhibitory concentrations (MICs) of the REO against the test organisms were determined by the micro-dilution method [35,36]. All reagents and materials used were placed in a biosafety cabinet. A 100 µL volume each of Mueller-Hinton broth was dispensed into all the 96 wells of the microtitre plates. Aliquots of 100 μL of REO were added in triplicate for each bacterial plate on the first rows (Row A). These were then serially diluted with 100 µL of each of the mixtures discarded from the last row, thus leaving each diluted well with a volume of 100 µL. This was followed by the addition of 100 µL of each of the 0.5 McFarland standardised test organisms on each column, except the last columns (12th wells), which served as the sterile controls (containing 200 µL of broth plus the diluent). The microtitre plates were then incubated in a shaking incubator at 37 °C for 24 h, and the MIC was depicted as the lowest concentration of REO that did not permit the growth of the test organism by the addition of 20 µL of MTT (3-(4,5-dimethylthiazole-2-yl)-2,5-diphenyltetrazolium bromide, 0.2%, *w*/*v*) to each well followed by incubation in a shaking incubator at 37 °C for 30 min. The MTT was prepared by measuring 0.1 g of the MTT dye and dissolving it in 100 mL of sterile distilled water. The least concentration of the mixture that did not show color change from yellow to purple was regarded as the MIC. The MIC was confirmed by spectrophotometry at 490 nm in a microtitre plate reader by recording absorbance values for the estimation of the IC50s [35,37].

In order to confirm if the REO was able to kill the microbial cells (bactericidal effect), the MBC was determined. Aliquots from each well from susceptibility testing assays were transferred to plates containing nutrient agar by streaking and then incubated for 24 h at 37 °C, after which the plates were checked for the presence or absence of growth in the nutrient agar [38].

### 4.11. Determination of REO Synergy with Conventional Antibiotics

Combinatory effects between the test REO and antibiotics were conducted using the checkerboard against the strains of test microbes with slight modification according to the method reported by da Silva et al. [39], Khodavandi et al. [40], and Dickson et al. [41]. Briefly, solutions with different proportions of each REO:drug (final volume of 200 μL) were prepared from twice the MIC solutions of each test sample and the individual antibiotics (1 mg/mL), and the antimicrobial activity was tested as described for MIC determination. The fractional inhibitory concentration index (FICI) was calculated according to the equation below:

$$FIC\;index=\frac{\left[MIC\;of\;antibiotic\;in\;combination\right]}{\left[MIC\;of\;antibiotic\;alone\right]}+\frac{\left[MIC\;of\;oil\;in\;combination\right]}{\left[MIC\;of\;oil\;alone\right]}$$

The interaction between the test essential oils and antibiotics was considered synergistic if the FIC index was ≤0.5, partial synergistic if the FIC index was >0.5 and <1, additive if the FIC index was =1, no difference if the FIC index was >1 and ≤4, and antagonistic if the FIC index was >4.0.

### 4.12. Determination of Antibiofilm Activity of REO

The antibacterial activities of the oil against microbial biofilms under two different treatment options (inhibition of biofilm formation and activity against preformed biofilms) were evaluated using the 96-well microtitre plate of microbial biofilm formation and susceptibility testing, with slight modifications [42,43]. Briefly, for the inhibition of biofilm formation, the double-strength Mueller-Hinton broth (Oxoid Limited, Basingstoke, UK) and the oil extract (100 µL each) were mixed in Column 1 and serially diluted until Column 10 at concentrations ranging from 0.04 to 20 mg/mL. Thereafter, 100 µL of the microbial suspension at a concentration of 1 × 10^6^ cells/mL was added to the wells of Columns 1–11 to arrive at a final volume of 200 µL. To each of these plates, 2 µL of 5% (*w*/*v*) sterile-filtered TTC solution (which was used in place of XTT/menadione solution) was added so that the final concentrations of TTC became 0.05% (*w*/*v*). The microtitre plates were immediately incubated for 24 h at 37 °C. On the other hand, to determine the inhibition against preformed biofilms, the microbial inoculums (100 µL of a suspension of 1 × 10^6^ cells/mL double-strength Mueller-Hinton broth) were pipetted into each well of a flat-bottom 96-well microplate and subjected to incubation at 37 °C for 24 h to allow for biofilm formation. Subsequently, the mixtures were carefully aspirated not to touch the preformed biofilm, which was then washed with PBS (100 µL) two times to remove planktonic and non-adherent cells. In another microplate, the dilutions of the free extract were prepared from 0.04 to 20 mg/mL and then transferred to the microtitre plate containing the preformed biofilms, which was incubated for an additional 24 h at 37 °C. Then, in the same vein, 2 µL of a TTC, 5% (*w*/*v*) sterile-filtered solution was added so that the final concentrations of TTC became 0.05% (*w*/*v*) and incubated. After the incubation periods, the liquid in each well of the microplates from both assays was carefully aspirated, and the biofilm was washed with PBS (100 µL) twice in order to remove planktonic and non-adherent cells. The post-processing to measure the metabolic activity after the antimicrobial (oil) treatment was evaluated as previously described [42,43], but by using the TTC reduction assay. Finally, plates were read by spectrophotometry at a modified OD of 492 nm in a microtitre plate reader, and the percentage microbial inhibition obtained by the equation below for the IC50s was subsequently calculated.

$$\%\;biofilm\;inhibition=\left(optical\;density\;\left(OD\right)\;of\;control-\frac{OD\;of\;treatment}{OD\;of\;control}\right)\times 100\%$$

## 5. Conclusions

Rosemary essential oil had significant bactericidal effects on the bacteria tested (including the multidrug-resistant ones), and its MIC and MBC values were low. Overall, REO was synergistic with known conventional antibiotics and, thus, has encouraging prospects in combination therapy involving conventional antibiotics, even in the treatment of infections with multidrug-resistant bacteria, biofilm-forming ones inclusive. Further authentication of the synergistic effects of REO is, nonetheless, warranted, and these investigations can expand the range of conventional antibiotics tested. Moreover, additional analyses on REO can clarify insights on its mechanism of action, as well as its potential acute and sub-chronic toxicity to humans and animals at therapeutic levels.

## Figures and Tables

**Table 1:** Major phytochemical constituents of the REO.

S/N	RT	Structure of Compound	Area %	MW/CID *	Molecular Formula	Common Name	IUPAC Nomenclature
1	8.037	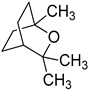	42.681	154 2758 *	C_10_H_18_O	Eucalyptol	(1S,4S)-1,3,3-trimethyl-2-oxabicyclo [2.2.2]octane
2	9.724	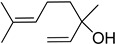	2.101	154 6549 *	C_10_H_18_O	Linalool	3,7-dimethylocta-1,6-dien-3-ol
3	9.944	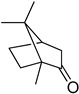	33.198	152 2537 *	C_10_H_16_O	(+)-2-Bornanone, camphor	(1S)-1,7,7-Trimethylbicyclo [2.2.1]heptan-2-one
4	10.641	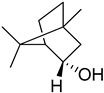	9.372	154 439,569 *	C_10_H_18_O	Endobornoel, camphol	(1S-endo)-1,7,7-Trimethylbicyclo [2.2.1]heptan-2-ol
5	10.714	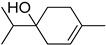	0.938	154 11,230 *	C_10_H_18_O	4-Terpineol, terpinen-4-ol	(R)-4-methyl-1-(1-methylethyl)-3-cyclohexen-1-ol
6	10.916	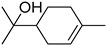	7.947	154 1700 *	C_10_H_18_O	α-Terpineol	2-(4-Methylcyclohex-3-en-1-yl)propan-2-ol
7	11.887	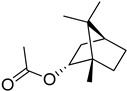	1.808	196 93,009 *	C_12_H_20_O_2_	Bornyl acetate	(1S,2R,4S)-1,7,7-trimethylbicyclo [2.2.1]heptan-2-yl acetate
8	12.052	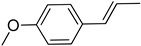	0.868	148 637,563 *	C_10_H_12_O	Anethole	(E)-1-methoxy-4-(prop-1-en-1-yl)benzene
9	13.758	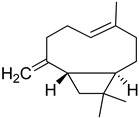	1.086	204 5,281,522 *	C_15_H_24_	Caryophyllene	(1R,4Z,9S)-4,11,11-trimethyl-8-methylenebicyclo [7.2.0]undec-4-ene

* = PubChem ID.

**Table 2:** Zones of inhibition (in millimeters) of different bacterial strains to rosemary essential oil.

Bacterial Strains	Concentration of Rosemary Essential Oil (mg/mL)					Control	
	200	100	50	20	10	Positive	Negative
*E. coli*	17.50 ± 1.32	16.67 ± 1.53	8.00 ± 1.00	9.00 ± 2.00	7.67 ± 1.53	25.50 ± 0.87	–
*K. pneumoniae*	18.50 ± 1.32	0.00 ± 0.00	0.00 ± 0.00	0.00 ± 0.00	0.00 ± 0.00	11.67 ± 0.58	–
MRSA	16.33 ± 1.15	14.67 ± 0.58	11.33 ± 1.15	11.00 ± 0.00	9.67 ± 0.58	30.33 ± 1.15	–
*S. mutans*	11.67 ± 0.58	10.00 ± 0.00	9.67 ± 1.15	0.00 ± 0.00	0.00 ± 0.00	22.67 ± 1.15	–
*B. subtilis*	14.67 ± 0.58	13.00 ± 1.15	13.33 ± 1.15	10.67 ± 1.15	9.33 ± 1.15	24.67 ± 1.15	–
*P. aeruginosa*	13.00 ± 0.00	12.67 ± 0.58	11.33 ± 1.15	10.67 ± 0.58	10.00 ± 0.00	21.67 ± 0.58	–
*S.* Typhi	25.67 ± 2.52	16.83 ± 2.02	12.33 ± 0.58	7.67 ± 1.15	5.50 ± 0.50	39.33 ± 0.58	–

Positive control = 30 µg chloramphenicol; Negative control = 20% DMSO in 0.5% Tween 80

**Table 3:** Minimum inhibitory effect of rosemary essential oil and antibiotics on different bacterial strains.

Bacterial Strains	Concentration of REO			CIP	CHL	STR	TET	AMP
	MIC (mg/mL)	MBC (mg/mL)	R	MIC (µg/mL)	MIC (µg/mL)	MIC (µg/mL)	MIC (µg/mL)	MIC (µg/mL)
*E. coli*	6.25	6.25	1	0.98	3.92	1.96	7.81	250
*K. pneumoniae*	6.25	6.25	1	1.25	7.81	125	7.81	1000
MRSA	6.25	12.5	2	62.5	7.81	3.91	7.81	250
*S. mutans*	6.25	12.5	2	125	15.68	250	31.25	1000
*B. subtilis*	3.13	3.13	1	125	15.68	250	31.25	1000
*P. aeruginosa*	3.13	3.12	1	125	31.25	500	31.25	1000
*S.* Typhi	3.13	6.25	2	0.98	15.68	125	1.96	1000

REO = Rosemary essential oil, MIC = minimum inhibitory concentration, MBC = minimum bactericidal concentration, R = ratio of MBC to MIC, R ≤ 4 = bactericidal, R > 4 = bacteriostatic, CIP = ciprofloxacin, CHL = chloramphenicol, STR = streptomycin, TET = tetracycline, AMP = ampicillin; details of the interpretative criteria can be found in the EUCAST guidelines.

**Table 4:** Synergistic effect of rosemary essential oil on antibiotics against different bacterial strains.

Bacterial Strain	FICI		FICI		FICI		FICI		FICI	
	CIP + REO	INT	CHL + REO	INT	STR + REO	INT	TET + REO	INT	AMP + REO	INT
*E. coli*	1.00	I	1.03	I	16.07	A	40.19	A	0.25	S
*K. pneumoniae*	0.79	PS	1.06	I	0.37	S	4.13	A	0.16	S
MRSA	0.31	S	2.12	I	8.12	A	0.62	PS	0.25	S
*S. mutans*	0.37	S	2.12	I	0.25	S	1.12	I	0.16	S
*B. subtilis*	0.19	S	2.12	I	0.25	S	1.13	I	0.16	S
*P. aeruginosa*	0.19	S	0.56	PS	0.19	S	1.13	I	0.16	S
*S.* Typhi	1.03	I	1.06	I	0.37	S	8.10	A	0.16	S

FICI = Fractional inhibitory concentration index, FICI ≤ 0.5 = synergism (S), FICI > 0.5 and < 1 = partial synergism (PS), FICI = 1 = additive (AD), FICI > 1 and ≤ 4 = indifference or no difference (I), FICI > 4 = antagonism (A), INT = interpretation.

**Table 5:** IC50 values for inhibition of pre-biofilm and biofilm formation of different bacterial strains by rosemary essential oil.

Bacterial Strains	IC50 (µg/mL)	
	Pre-Biofilm	Biofilm
*E. coli*	1342.00	4.00
*K. pneumoniae*	106.00	3.00
MRSA	134.00	6.00
*S. mutans*	7259.00	7.00
*B. subtilis*	120.00	7.00
*P. aeruginosa*	4989.00	7.00
*S.* Typhi	10.00	2.00

## Data Availability

All the relevant data are contained in the manuscript.

## Supplementary Materials

The following supporting information can be downloaded at: https://www.mdpi.com/article/10.3390/ph17121653/s1, Figure S1.Zones of Inhibition (in millimeters) of different bacterial strains to Rosemary essential oil; Figure S2. The GC/MS analysis of Rosemary essential oil (REO), Figure S3. The total ion chromatogram (TIC) or mass chromatogram) of Eucalyptol: Scan time = 8.037 min; Figure S4. The total ion chromatogram (TIC) or mass chromatogram) of Linalool: Scan time = 9.430 min; Figure S5. The total ion chromatogram (TIC) or mass chromatogram) of (+)-2-Bornanone: Scan time = 9.944 min; Figure S6. The total ion chromatogram (TIC) or mass chromatogram) of Endo-Borneol: Scan time = 10.641 min; Figure S7. The total ion chromatogram (TIC) or mass chromatogram) of Terpinen-4-ol: Scan time = 10.714 min; Figure S8. The total ion chromatogram (TIC) or mass chromatogram) of α-Terpineol: Scan time = 10.916 min; Figure S9. The total ion chromatogram (TIC) or mass chromatogram) of Bornyl acetate: Scan time = 11.887 min; Figure S10. The total ion chromatogram (TIC) or mass chromatogram) of Anethole: Scan time = 12.052 min; Figure S11. The total ion chromatogram (TIC) or mass chromatogram) of Caryophyllene: Scan time = 13.758 min.
