# Supplementary material for: In Vitro Evaluation of Rosemary Essential Oil: GC-MS Profiling, Antibacterial Synergy, and Biofilm Inhibition
Source: Pharmaceuticals (Basel). 2024 Dec 8;17(12):1653. doi: 10.3390/ph17121653 (PMC11728608; doi:10.3390/ph17121653)
Supplement: Supplementary file 1 [file pharmaceuticals-17-01653-s001.zip › pharmaceuticals-3249136-supplementary.pdf]

# Supporting Information

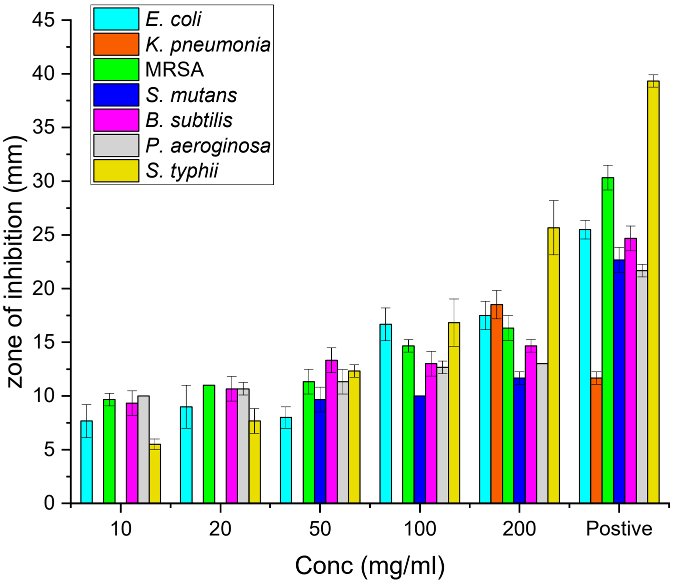


**Figure S1.** Zones of Inhibition (in millimeters) of different bacterial strains to Rosemary essential oil

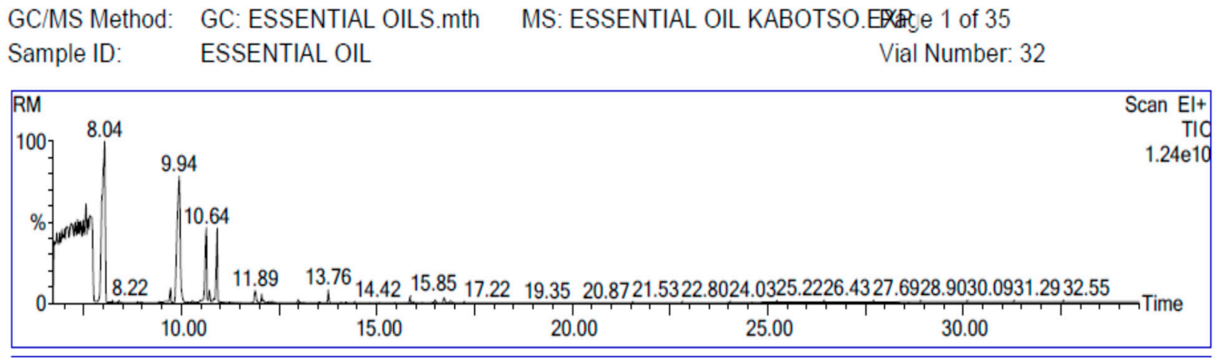


**Figure S2.** The GC/MS analysis of Rosemary essential oil (REO).

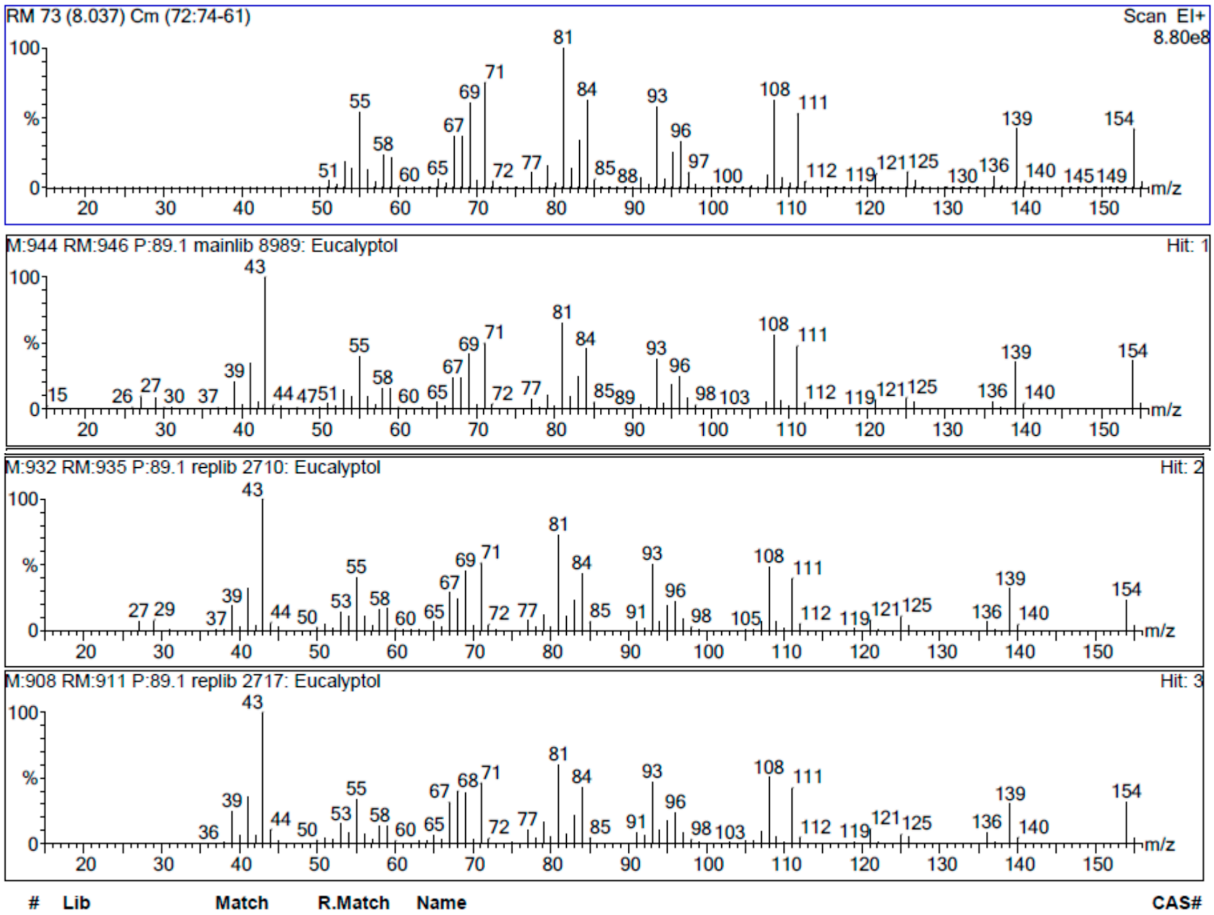


**Figure S3**. The total ion chromatogram (TIC) or mass chromatogram) of **Eucalyptol**: Scan time = 8.037 min

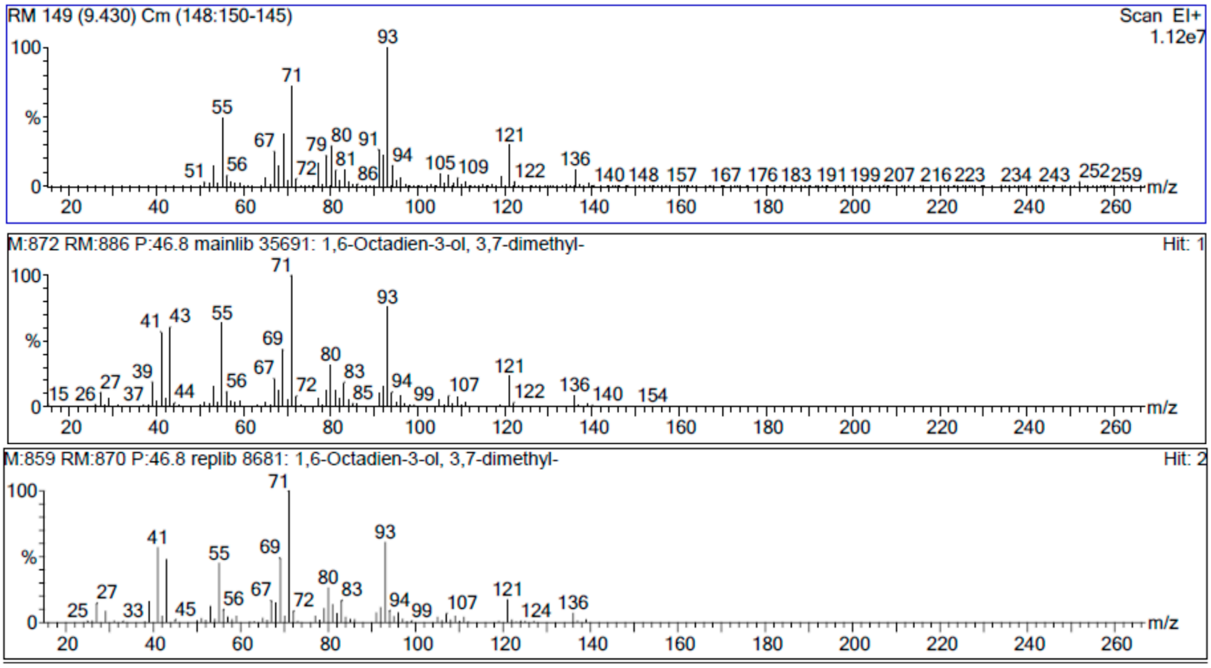


**Figure S4**. The total ion chromatogram (TIC) or mass chromatogram) of **Linalool**: Scan time = 9.430 min

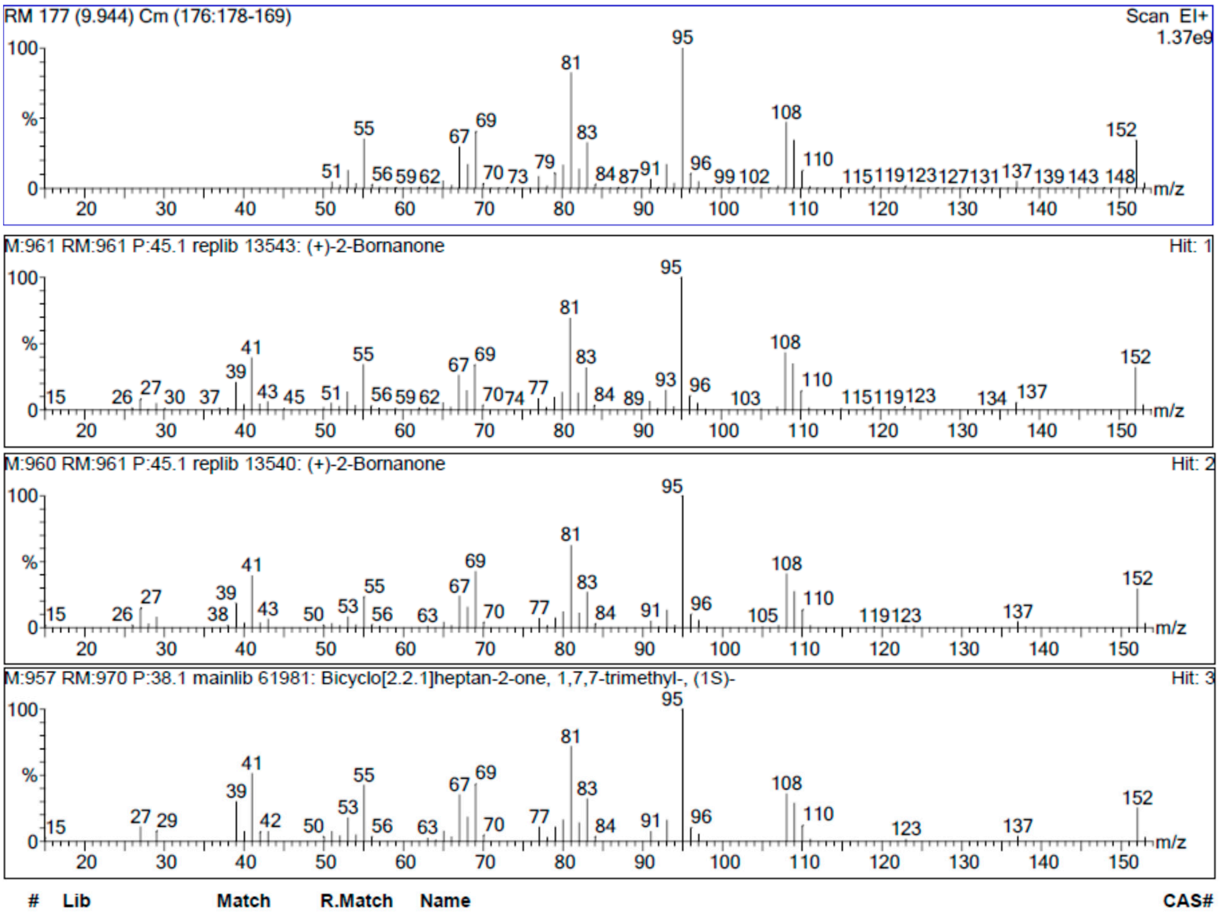


**Figure S5**. The total ion chromatogram (TIC) or mass chromatogram) of **(+)-2-Bornanone**: Scan time = 9.944 min

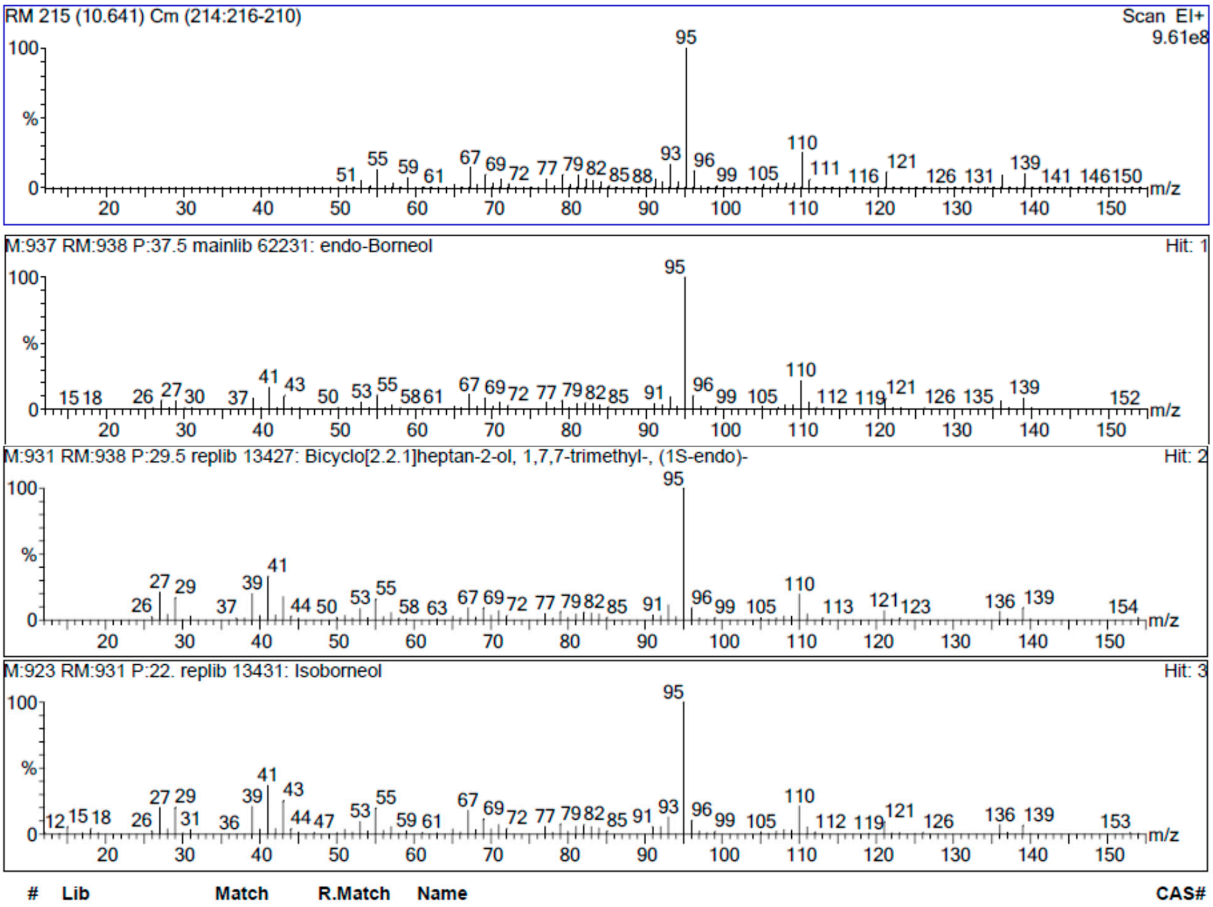


**Figure S6**. The total ion chromatogram (TIC) or mass chromatogram) of **Endo-Borneol**: Scan time = 10.641 min

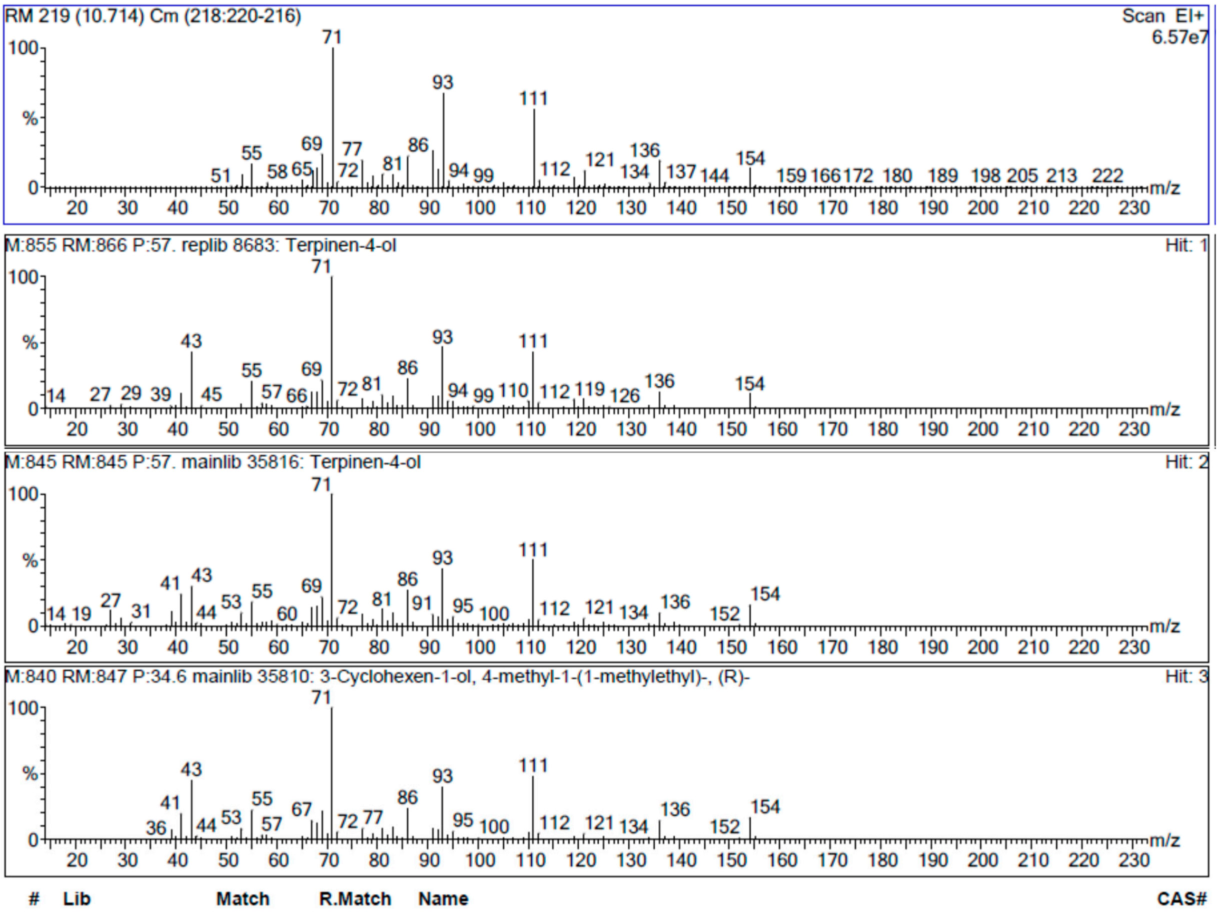


**Figure S7**. The total ion chromatogram (TIC) or mass chromatogram) of **Terpinen-4-ol**: Scan time = 10.714 min

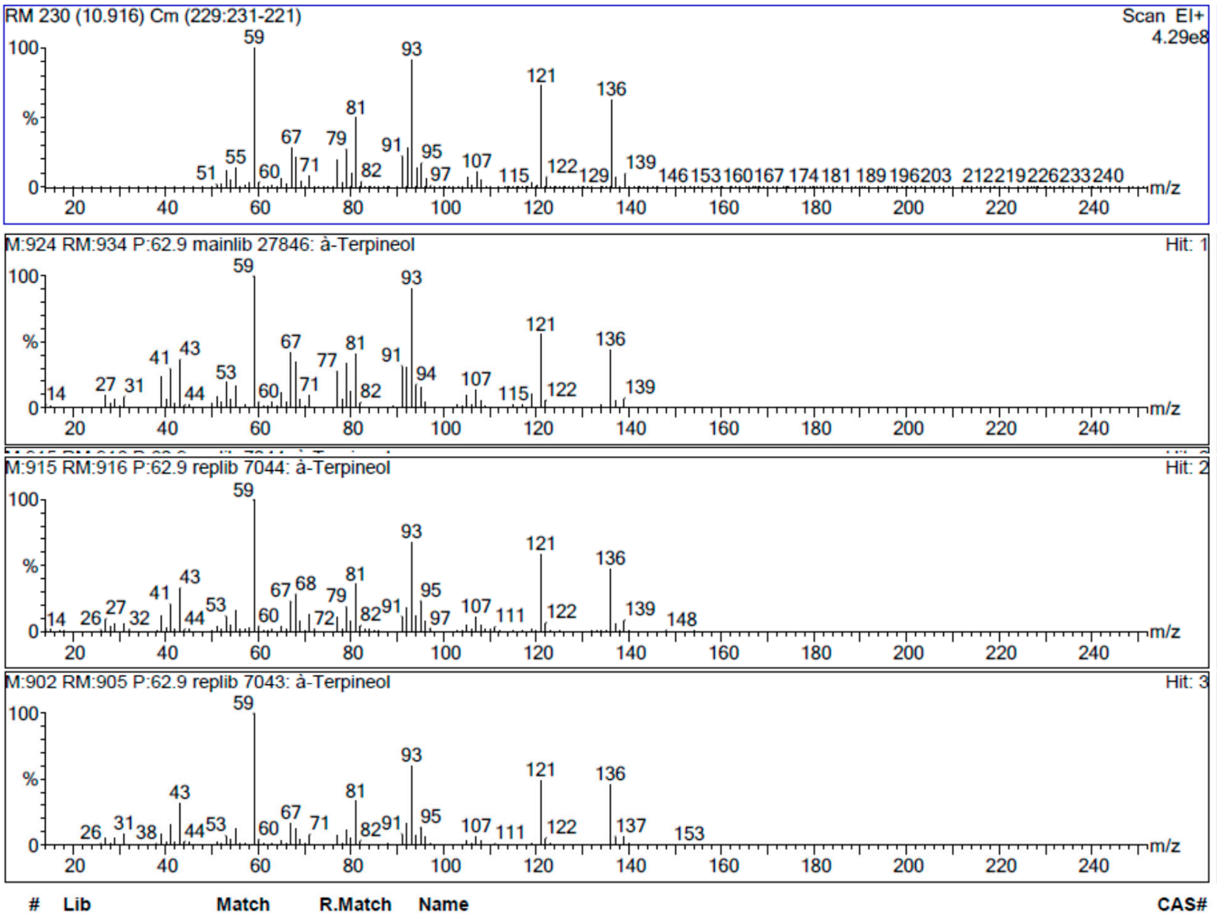


**Figure S8**. The total ion chromatogram (TIC) or mass chromatogram) of ***α*-Terpineol**: Scan time = 10.916 min

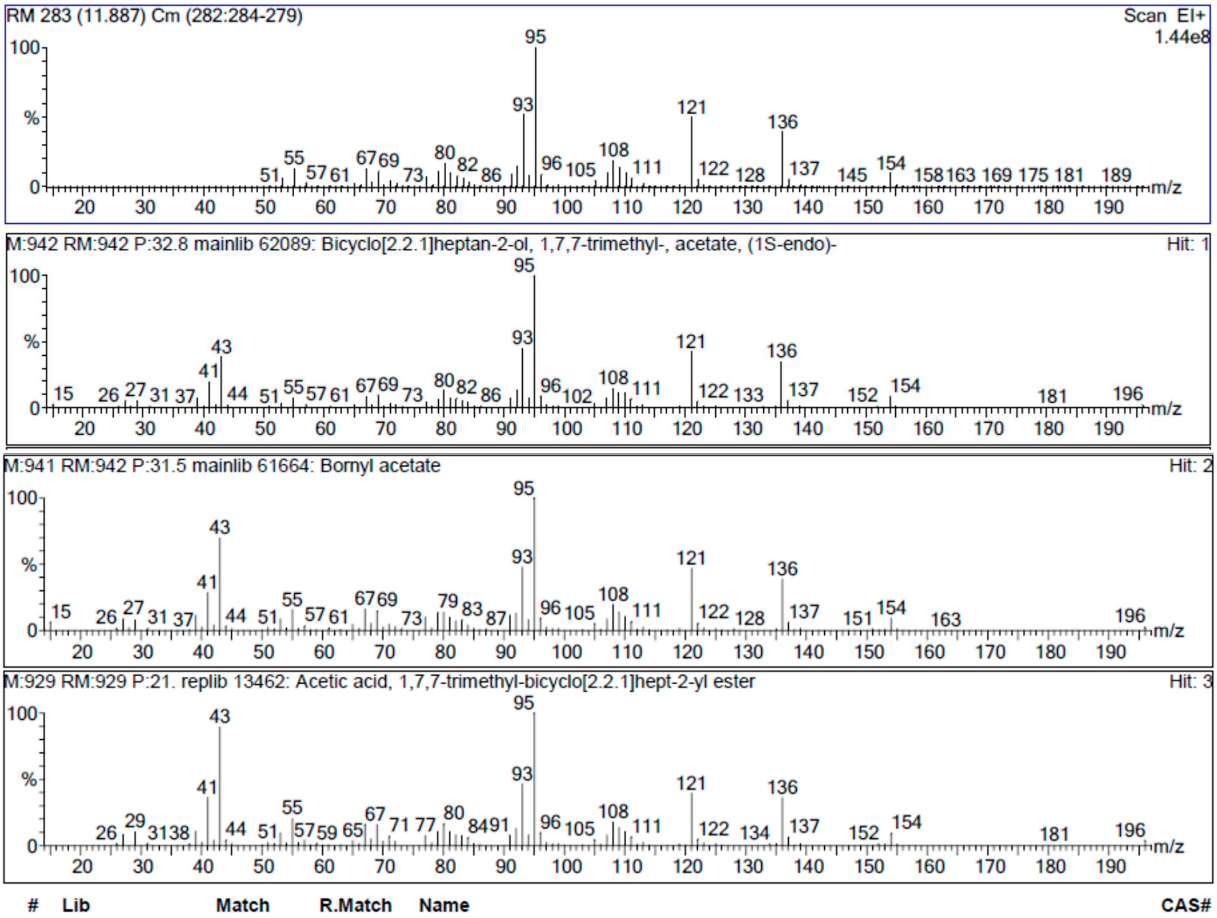


**Figure S9**. The total ion chromatogram (TIC) or mass chromatogram) of **Bornyl acetate**: Scan time = 11.887 min

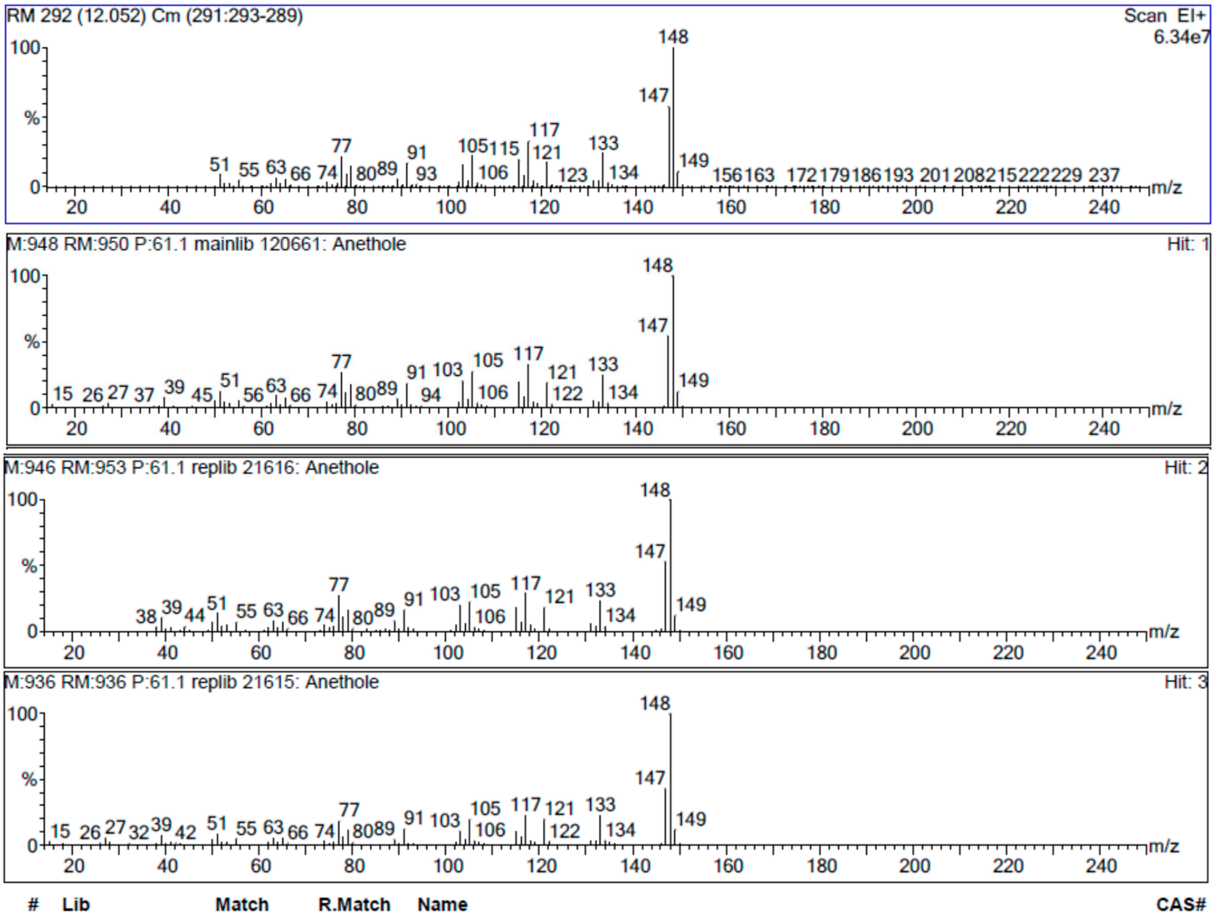


**Figure S10**. The total ion chromatogram (TIC) or mass chromatogram) of **Anethole**: Scan time = 12.052 min

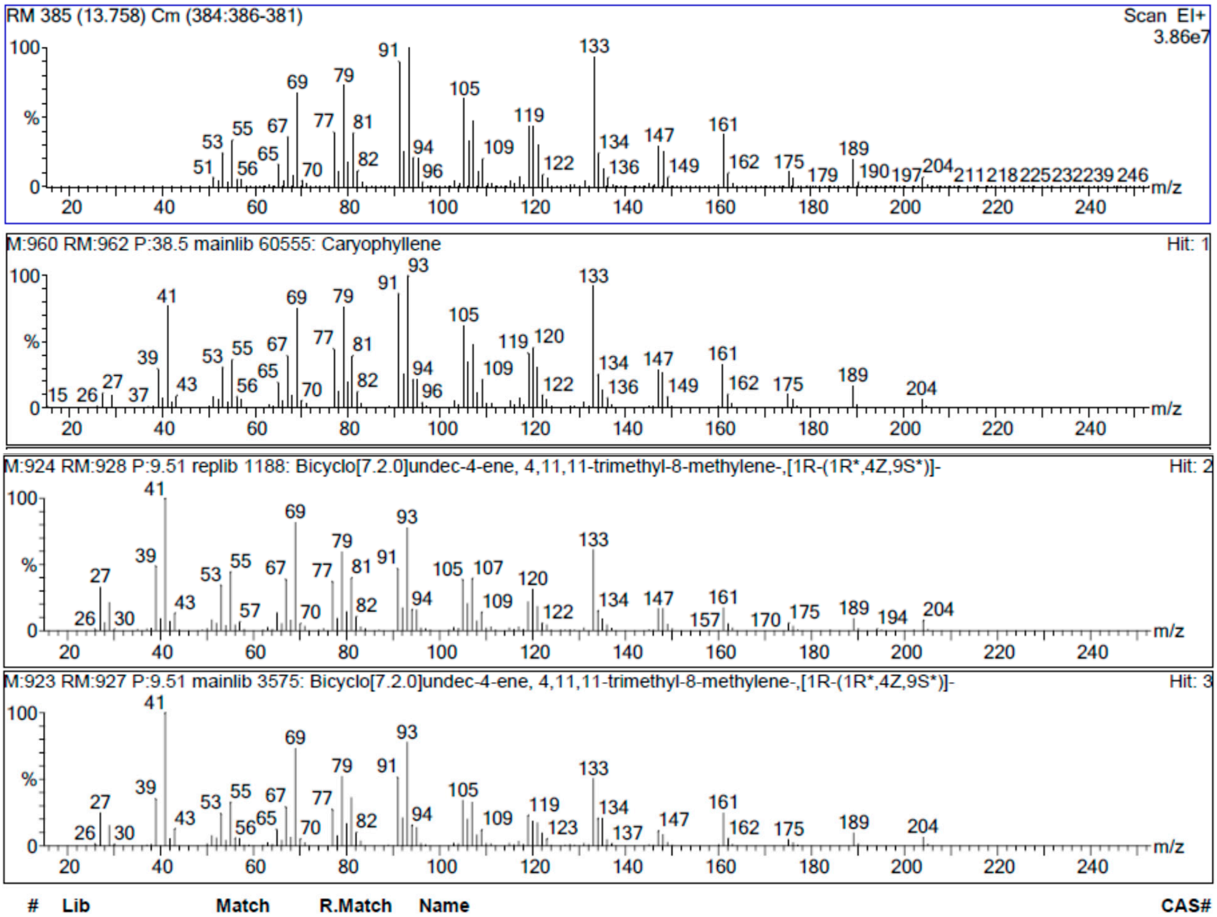


**Figure S11**. The total ion chromatogram (TIC) or mass chromatogram) of **Caryophyllene**: Scan time = 13.758 min
